# New Records of *Isognomon* Species from Crete, Greece; Evidence from Adult Specimens and Additional DNA Barcodes

**DOI:** 10.3390/ani16142277

**Published:** 2026-07-22

**Authors:** Papavasileiou Kyriakos, Giagkazoglou Zoi, Karagianni Athanasia, Sidiropoulou Dimitra, Loukovitis Dimitrios, Imsiridou Anastasia, Manousis Thanasis, Galinou-Mitsoudi Sofia

**Affiliations:** 1 Independent Researcher, 55131 Kalamaria, Greece; kpapav@yahoo.com; 2 Department of Food Science and Technology, School of Geosciences, International Hellenic University, 57400 Thessaloniki, Greece; athanasiakaragianni02@gmail.com (K.A.); dimitra.sidiropoulou2001@gmail.com (S.D.); imsiri@otenet.gr (I.A.); 3 Department of Fisheries and Aquaculture, School of Agricultural Sciences, University of Patras, 26504 Patras, Greece; dloukovi@upatras.gr; 4 Independent Researcher, 57500 Epanomi, Greece; athanasiosmanousis@gmail.com; 5 Department of Environmental Engineering, School of Engineering, International Hellenic University, 57400 Thessaloniki, Greece; galimits@ihu.gr

**Keywords:** adult morphometrics, *COI*, eastern Mediterranean, *Isognomon bicolor*, *16S rRNA*, *28S rRNA*, phylogeny, taxonomy

## Abstract

During the last two decades, non-indigenous species of the genus *Isognomon* have been increasingly reported in the Mediterranean Sea, yet their taxonomy and distribution remain poorly resolved because of pronounced shell plasticity and limited molecular reference data. In the present study, *Isognomon* specimens collected along the coasts of Crete (Greece) were examined, using an integrative approach combining adult morphometric analyses with mitochondrial (*COI* and *16S rRNA*) and nuclear (*28S rRNA*) genetic markers. The results confirm the establishment of *Isognomon bicolor* in the eastern Mediterranean and document the presence of specimens morphologically consistent with *I. australicus*, which clustered within the unresolved *I. legumen–I. aff. legumen* complex in both mitochondrial and nuclear phylogenetic analyses. Morphometric analyses revealed pronounced shell plasticity, with shell width, hinge length, and nacreous height providing the greatest discriminatory power, although considerable overlap between taxa limited reliable identification based solely on shell morphology. Molecular analyses confirmed the identity of *I. bicolor* and further supported its eastward expansion within the Mediterranean basin. In contrast, the concordance between mitochondrial and nuclear datasets indicates that the unresolved taxonomy of the *I. australicus–I. legumen* complex is unlikely to result solely from the use of mitochondrial markers. These findings expand the available morphometric and genetic data for Mediterranean *Isognomon* populations and highlight the need for integrative taxonomic approaches, incorporating additional nuclear and genomic data to resolve species boundaries.

## Simple Summary

Marine species that originate outside the Mediterranean Sea are increasingly appearing along its coasts as sea temperatures rise and human activities, particularly shipping, transport organisms between regions. Among them are oysters of the genus *Isognomon*, which attach to rocky shores and artificial structures and may influence local marine ecosystems. However, identifying these species is challenging because their shells vary considerably depending on environmental conditions. In this study, we examined *Isognomon* specimens collected from the coasts of Crete, Greece, using both shell measurements and genetic analyses. We confirmed the establishment of the non-native oyster *Isognomon bicolor* and recorded specimens belonging to a closely related species complex, whose exact identity remains unresolved despite the use of both mitochondrial and nuclear genetic markers. Our findings provide new information on the distribution and taxonomy of *Isognomon* in the eastern Mediterranean and highlight the importance of combining morphological and molecular approaches when studying non-native marine species. Improving knowledge of their identity and distribution will help scientists better understand changes in marine biodiversity and support the conservation and management of Mediterranean coastal ecosystems.

## 1. Introduction

The genus *Isognomon* [Lightfoot], 1786 is a group of oysters belonging to the family of Isognomonidae Woodring, 1925 (1828), within the superfamily Pterioidea J. E. Gray, 1847 (1820) (WoRMS) (Web Resource 1). This group is primarily defined by shell morphology and ligament structure [1], with Isognomonidae distinguished by its characteristic multivincular ligament [2]. Their shells are highly irregular in shape and possess a pearly interior [3]. More specifically, the shell shape and thickness may indicate hydrodynamic effects and nutritional conditions [4]. Like many other bivalves, members of this genus function as key ecosystem engineers, enhancing habitat complexity, contributing to water filtration, and supporting marine food webs through both structural and trophic roles [5].

The establishment of non-indigenous species (NIS) in the Mediterranean is becoming increasingly common [6,7], driven by a combination of environmental, ecological, and anthropogenic factors [7]. Their successful establishment has been primarily linked to rising sea surface temperatures (SST) associated with global warming [8]. As a result, tropical and subtropical species that previously could not survive in the cooler Mediterranean waters now encounter more favorable conditions for settlement and expansion [7]. This trend is projected to intensify in the coming decades, as the Mediterranean is currently recognized as the fastest-warming sea worldwide [9], accompanied by a notable increase in warm-water species introductions, with nearly 1000 alien taxa now recorded in the basin [10]. Additionally, Lessepsian migration through the Suez Canal represents one of the principal pathways facilitating the introduction of Indo-Pacific species into the Mediterranean Sea [8]. Direct human activities such as hull fouling, untreated ballast water discharge, and the transport of organisms on floating macrolitter further contribute to their spread [11,12]. Changes in current regimes and circulation dynamics within the basin may also promote the dispersal of NIS to new regions, combining with human-mediated vectors and natural range expansions to enhance their spread [7]. By 2022, a total of 173 non-indigenous molluscan species were considered established in the Mediterranean Sea, an increase of approximately 38% over the past two decades [10]. Given this accelerating introduction and establishment of alien marine taxa, increasing attention has been directed toward species of the genus *Isognomon*, which have recently expanded into the Mediterranean Sea. Most published records concern *Isognomon bicolor* (C. B. Adams, 1845)*,* reported along the coasts of Israel, the Dodecanese Islands in Greece, and southern Italy [13], including recent observations of abundant populations in Sicily [14]. Consistent with this invasive potential, recent records show that members of the genus are now increasingly appearing beyond their native ranges, including the Mediterranean Sea [7,13]. In contrast, *Isognomon ephippium* (Linnaeus, 1758) has only been documented once in the Mediterranean, recorded on an oil platform transported from Australia to Israel, a finding considered incidental rather than indicative of establishment [15,16].

Species of the genus *Isognomon* have a pan-tropical distribution and are among the most common mollusks inhabiting intertidal and infralittoral zones across the Indian, Atlantic, and Pacific Oceans, with their highest diversity reported in the Indo-West Pacific [17,18]. They occupy a wide range of substrates, including rocky shores, mangroves, coral reefs, sandy coastlines, algal turf, floating debris, and various artificial structures [18]. Such ecological versatility indicates a high degree of environmental adaptability, a trait also recognized in other mollusks capable of successfully adjusting to a broad spectrum of habitat conditions [19]. This adaptability is reflected in the fact that several *Isognomon* species have been reported as invasive outside their native ranges, with *I. bicolor* serving as a prominent example, having established and expanded across multiple regions of the Brazilian coastline [20,21,22]. 

During the past two decades, members of the genus have been increasingly reported from the Mediterranean region, including *Isognomon australicus* [23,24,25], *I. bicolor* [14,26], *I. ephippium* (Linnaeus, 1758) [15], and *Isognomon legumen* (Gmelin, 1791) [15,23,27], suggesting a distribution extending from the Eastern to the Central Mediterranean Sea. Recent publications have instead adopted the names *Isognomon australica* (Reeve, 1858) (sensu Angelidis and Polyzoulis 2018) [23] and *I. legumen* [13] in place of *I. australicus* [23], highlighting ongoing taxonomic uncertainty and the need for detailed molecular and ecological investigation [28].

Taxonomic ambiguity is particularly severe within the bivalve class due to the high phenotypic plasticity of shells [29]. Species of the genus *Isognomon* exhibit substantial morphological and ecological variability—including marked differences in size, shell geometry, and habitat preference—and occur across three major ocean basins [7,18]. Currently, the genus is represented worldwide by 16 accepted species [18]. However, their diagnostic characteristics remain problematic, as species are mostly distinguished by descriptive conchological traits such as shell outline, obliquity, auricle development, and variations in shape and color [7,30,31]. This challenge is further intensified by environmentally driven variation in shell morphology, where even small differences in habitat conditions can result in misleading phenotypic changes [32]. As a result, closely related or cryptic species may remain indistinguishable based solely on shell characters [18]. This complexity has led, for example, to frequent confusion between *Isognomon alatus* (Gmelin, 1791) and *I. bicolor* in the field, as well as misidentifications of *I. alatus* as *I. ephippium* in museum collections [32]. Similarly, *Isognomon spathulatus* has been mistakenly synonymized with *I. ephippium* due to overlapping shell morphologies [3,18,33].

For this reason, molecular approaches such as DNA barcoding and phylogenetic analyses provide an effective alternative when traditional taxonomy is inconclusive. However, the success of these methods depends on the availability of comprehensive and reliable reference libraries, including global databases like GenBank and BOLD [34]. A key limitation, however, is that many historical type specimens are shell-only and lack genetic material, making it difficult to confirm whether newly sequenced individuals correspond to the originally described taxa. This gap highlights the need for integrative approaches to reconcile morphological names with molecular data [17]. In addition, a considerable proportion of sequences stored in public databases may be inaccurately identified, leading to uncertainty in the molecular identification of molluscan taxa [26]. Mislabeling and taxonomic inconsistencies have been widely documented in GenBank and BOLD, affecting multiple families and reflecting the challenge of relying on reference material that is not consistently curated [35,36]. Recently, the systematics of the genus *Isognomon* in the Mediterranean Sea was revised using both morphological and molecular approaches and showed the presence of two species only: *I. bicolor*, distributed in the Central and Eastern Mediterranean, and the Indo-Pacific *I. australicus*, only from the Eastern Mediterranean, which was suggested to be renamed as *I. aff. legumen* [7,28,37].

Despite recent advances in the systematics of Mediterranean *Isognomon* populations [7,28,37], information from the eastern Mediterranean remains limited, particularly regarding adult morphometrics and the genetic diversity of Cretan populations. Here, we examined *Isognomon* specimens collected from Crete, Greece, providing new records of occurrence and establishment, quantitative morphometric data from adult individuals, additional *COI* and 16S haplotypes, and nuclear 28S sequences. These findings expand the available morphological and genetic knowledge of eastern Mediterranean *Isognomon* populations and provide further evidence for evaluating their taxonomic affinities.

## 2. Materials and Methods

### 2.1. Morphological Examination and Measurements

#### 2.1.1. Sampling and Morphological Identification

**Figure 1:**
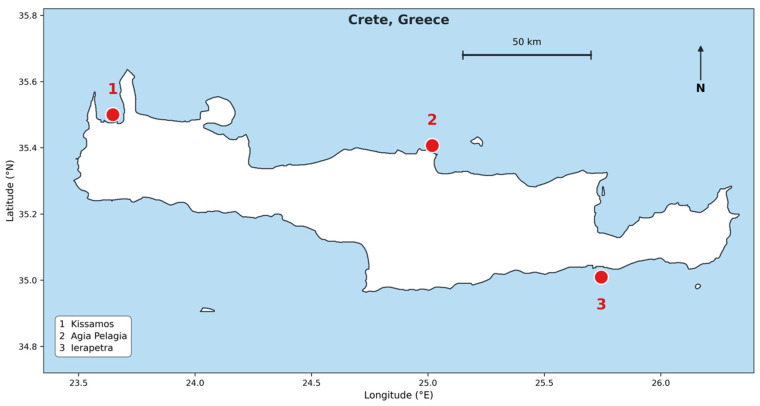
The collection locations. 1. Kissamos, Chania; 2. Agia Pelagia, Heraklion; 3. Ierapetra, Lasithi.

A total of 40 *Isognomon* specimens were collected in August 2023 from three locations in Crete (Figure 1). Sampling was conducted through in situ snorkeling surveys, during which specimens were carefully retrieved from rock crevices and under stones, with care taken to avoid shell damage and minimize potential effects of rocky substrates, hydrodynamism, and swelling [30]. The sampling sites included Kissamos, Chania (35°30.00′ N, 23°38.85′ E), Agia Pelagia, Heraklion (Northern Crete; 35°24.40′ N, 25°01.13′ E), and Ierapetra, Lasithi (Southern Crete; 35°00.56′ N, 25°44.67′ E). All samples were preserved in 95% ethanol until further analysis.

**Figure 2:**
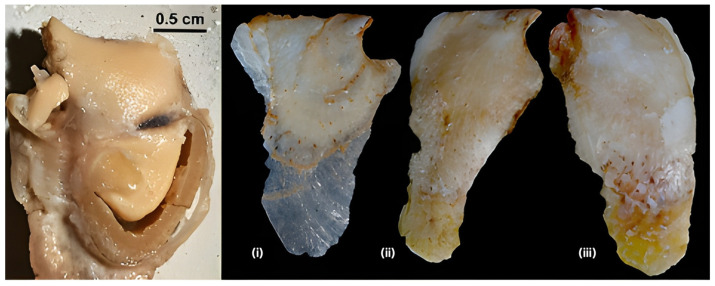
Specimens morphologically identified as *I. australicus*. (**Left**): Body showing the adductor muscle. (**Right**): Brown spots as visible on the external surfaces of the valves: (**i**) left and (**ii**) right valves; (**iii**) left valve of the same specimen shown in (**ii**).

**Figure 3:**
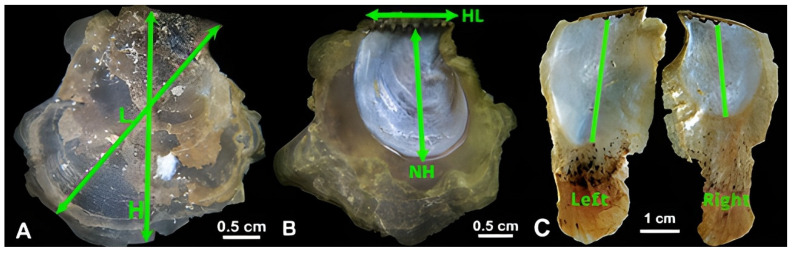
Dimensions. (**A**,**B**), *I. bicolor*; (**C**), specimens morphologically identified as *I. australicus*; nacreous heights of valves. (L) length, (H) height, (HL) hinge length, (NH) nacreous height.

Specimens were morphologically identified as *I. australicus* and *I. bicolor* based on external and internal morphological characters. Identification of *I. australicus* (Figure 2) relied primarily on the presence of small brown orthogonal spots arranged along weak radial ribs (Figure 2 and Figure 3C), following published species figures (Web Resources 1 and 2). Valve scars were also examined as additional diagnostic features [22,37] (Web Resource 2). Species nomenclature followed WoRMS (Web Resource 1). Observations were performed using a Zeiss Stemi-C stereomicroscope (Zeiss, Oberkochen, Germany) with magnification up to 125×. *I. bicolor* was identified based on detailed diagnostic features described by [21].

#### 2.1.2. Measurements

Biometric measurements were obtained from all specimens corresponding to *I. bicolor* and specimens morphologically identified as *I. australicus* collected in August 2023, a period representing peak summer conditions in the Mediterranean Sea, during which seawater temperatures may reach approximately 30 °C (Web Resource 3). Shell shape was assessed to evaluate potential environmental pressures based on the direction of shell growth in relation to the byssus opening (anterior side) and categorized using capital letters as follows: posterior growth (J), anterior growth (L), and no directional change (straight; JL).

Size measurements were conducted on undamaged individuals, including length (L), height (H) (Figure 3A), width (W; maximum distance between the two valves), hinge length (HL) (Figure 3B), and nacre height (NH; right and left valves) (Figure 3B,C), all recorded with an accuracy of 0.01 cm. It is noted that the nacreous heights of *I. bicolor* are similar between the left and right valves whereas in specimens morphologically identified as *I. australicus* the nacreous area and height of the left valve are more extended compared to the right valve (Figure 3C). The hinge length (HL) and nacre height (NH right/left) are considered more reliable measurements, as they appear less affected by environmental pressures [38]. In addition, the number of hinge resilifers was recorded.

Weight measurements were performed on all intact individuals, including total weight (TWw), body weight (BWw), and shell weight (ShWw), expressed as wet weight (Ww) with an accuracy of 0.001 g. Shell growth was assessed indirectly, as the size of isognomonids appears to be related to the number of hinge resilifers, providing an indication of growth [39] (Web Resource 4). Due to the limited number of individuals, the polymorphism of shell shapes and the restricted size variation within the short sampling period, only the most significant relationships between biological parameters were considered, specifically those with *p* (ANOVA) < 0.05 and R^2^ > 80% (e.g., shell dimensions and hinge characteristics). To evaluate morphological differentiation between species, one-way analysis of variance (ANOVA) was performed for the shell morphometric variables. Variables showing significant differences were visualized using boxplots and subsequently used in a Principal Component Analysis (PCA) to assess patterns of morphological variation and species separation. Statistical significance was set at *p* < 0.05.

### 2.2. Genetic Study

#### 2.2.1. DNA Extraction, Amplification and Sequencing

The adductor muscle was removed from twenty *I. bicolor* specimens and sixteen specimens morphologically identified as *I. australicus*, preserved in absolute ethanol, and used for DNA extraction. DNA was extracted using the DNeasy Blood & Tissue Kit (Qiagen, Hilden, Germany), according to the manufacturer’s instructions. The extracted DNA was visualized on a 1% agarose gel stained with Midori Green DNA dye (Nippon Genetics Europe GmbH, Düren, Germany), purified using the DNA Clean & Concentrator kit (ZYMO Research, Tustin, CA, USA), and stored at −20 °C until further analysis.

The universal primer set 16S-L/16S-H designed by Palumbi [40] was used for the amplification of a 600 bp fragment of the mtDNA *16S rRNA* gene in both species. PCR was conducted in a total volume of 25 μL using the KAPA2G Robust HotStart ReadyMix (KAPA Biosystems, Cape Town, South Africa). The reaction mixtures contained 1 μL of template DNA, 1.25 μL of each primer (10 μM), 12 μL PCR mix, and 9.5 μL PCR-grade water. PCR cycling conditions followed the manufacturer’s recommendations, with an initial denaturation at 95 °C for 2 min, followed by 30 cycles at 94 °C for 30 s, 55 °C for 30 s, and 72 °C for 1 min, and a final extension at 72 °C for 10 min. Negative controls were included in all PCR runs. Electrophoresis of 5 μL of the PCR product was performed in 1 × TBE buffer (Applichem-Panreac, Darmstadt, Germany) for 1 h at 150 V on a 1.5% agarose gel (Applichem-Panreac, Germany), stained with Midori Green (Nippon Genetics, Düren, Germany). A FastGene 100 bp DNA Ladder (Nippon Genetics Europe GmbH, Germany) was loaded to verify the size of the PCR products. After electrophoresis, the resulting DNA fragments were visualized under UV transillumination and photographed.

PCR products were sequenced at the International Hellenic University (Department of Agriculture, Greece) using an ABI 3500 Genetic Analyzer (Applied Biosystems, Carlsbad, CA, USA). PCR products were sequenced in both the forward and reverse directions using the corresponding amplification primers. Forward and reverse reads were assembled into consensus sequences with BioEdit v7.2.6. and the resulting chromatograms were visually inspected and edited with the same program. Low-quality regions and ambiguous base calls were manually checked and edited prior to species identification analyses. Following sequencing, the *16S rRNA* sequences of *I. bicolor* individuals showed 98% similarity with *I. bicolor* sequences from GenBank, whereas the *16S rRNA* sequences obtained from specimens morphologically identified as *I. australicus* did not correspond to *Isognomon* species sequences after comparison using the BLAST engine. For this reason, the universal primer set jgLCO1490/jgHCO2198 of Geller [41] was used for the amplification of a 700 bp fragment of the mtDNA *COI* gene, in all specimens morphologically identified as *I. australicus*. PCR reagents, reaction mixtures, cycling conditions, and agarose gel electrophoresis conditions were the same as those used for the *16S rRNA* gene. To provide an additional line of nuclear molecular evidence for the taxonomic assessment of the specimens, two candidate markers, *28S rRNA* and ITS1, were initially considered. However, because reference sequences for *Isognomon* species were available only for the *28S rRNA* gene, a fragment of this marker was amplified using the primer pair D1R/D2C [42], despite its relatively slow evolutionary rate and potentially limited resolving power among closely related species. PCR amplification and sequencing were performed following the same procedures described above. Furthermore, the universal primer set 16Sar-L/16Sbr-H of Palumbi [43] was used to amplify a 600 bp fragment of the mtDNA *16S rRNA* gene in two specimens morphologically identified as *I. australicus* under the same conditions.

#### 2.2.2. Sequences Analysis and Comparison with the Databases

The obtained *COI*, *16S rRNA* and *28S rRNA* sequences were edited and aligned using the Clustal X software v2.0 [44] and the BioEdit software v7.2.6 [45]. All sequences were compared with those available in GenBank using the standard nucleotide BLAST (blastn) against the nucleotide collection (nr/nt) database (Web Resource 5). In addition, the *COI* sequences were compared with the Barcode of Life Data System (Web Resource 6) using the Identification System (IDs). For both databases, a sequence identity threshold of 98% was applied for species-level identification [46,47].

#### 2.2.3. Phylogenetic Analysis

Phylogenetic relationships were reconstructed separately for the *COI*, *16S rRNA*, and *28S rRNA* datasets using Maximum Likelihood (ML) and Neighbor-Joining (NJ) methods implemented in MEGA12 [48]. For the ML analyses, the best-fit nucleotide substitution model for each dataset was selected using the model-selection function implemented in MEGA12. Branch support for both methods was assessed using 1000 bootstrap replicates [49]. The ML trees are presented in the main manuscript, whereas the corresponding NJ trees are provided as Supplementary Figures S1–S3. Reference sequences retrieved from GenBank were included in each analysis (Supplementary Tables S1–S3). *Pinna nobilis* Linnaeus, 1758 was used as the outgroup for the *COI* and *16S rRNA* trees, whereas *Malleus malleus* (Linnaeus, 1758) was used for the *28S rRNA* trees.

#### 2.2.4. Pairwise Genetic Distance Analysis

Pairwise genetic distances were calculated separately for the *COI*, *16S rRNA* and *28S rRNA* datasets using MEGA12 [48]. These analyses were performed to evaluate intra- and interspecific genetic divergence among the examined specimens and the reference sequences included in each dataset.

#### 2.2.5. Genetic Diversity and Haplotype Analysis

Genetic diversity analyses were performed using R version 4.4.2 [50] and the pegas package v1.4 [51]. Sequence data were imported and handled using the ape package v5.8.1 [52]. The number of haplotypes (H), haplotype diversity (Hd), nucleotide diversity (π), and number of segregating sites (S) were calculated from the *28S rRNA* dataset for each sampling location. A haplotype network was reconstructed from the *COI* dataset using pegas [51], based on the statistical parsimony algorithm, to visualize the relationships among the identified haplotypes.

## 3. Results

### 3.1. Morphological Study

#### 3.1.1. Occurrence and Habitat Characteristics

*Isognomon* individuals were found aggregated in clusters of numerous specimens, settled on large rock fissures/crevices and under stones. The main ecological characteristics and the number of specimens per species in each study area were recorded as follows: (i) North Crete, Kissamos (Figure 1, station 1): depth −3 m; rocky substrate of low hydrodynamics covered with crustose algae in the upper sublittoral zone, followed at greater depth by dense *Cystoseira* sp. meadows; 4 specimens of *I. bicolor* and 6 specimens morphologically identified as *I. australicus* were collected. (ii) North Crete, Agia Pelagia (Figure 1, station 2): depth −2 m; bare rocky substrate dominated by sea urchins; 11 specimens of *I. bicolor* and 9 specimens morphologically identified as *I. australicus* were collected. (iii) South Crete, Ierapetra (Figure 1, station 3): exposed beach to southern winds; depth −4 m; mixed substrate of coarse gravel and stones, without macroalgae, under high hydrodynamic conditions; 6 specimens of *I. bicolor* and 4 specimens morphologically identified as *I. australicus* were collected.

#### 3.1.2. Biometric Characteristics and Statistical Analysis

The collected specimens of each *Isognomon* species from all locations were adults, and their measured biometric parameters are presented in Table 1. Mean values of most biometric parameters were generally higher for specimens morphologically identified as *I. australicus* than for *I. bicolor*, with the most pronounced differences observed in shell height (H) and the nacreous height (NH) of the left valve. More specifically, the nacreous layer height of the left valve is similar to that of the right valve in *I. bicolor*, whereas it is more extended in specimens morphologically identified as *I. australicus* (Figure 3C; Table 1).

**Figure 4:**
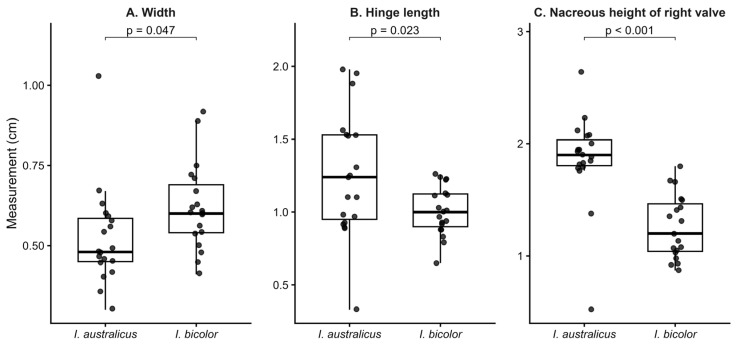
Boxplots of the three shell morphometric characters that differed significantly between *I. bicolor* and specimens morphologically identified as *I. australicus*: (**A**) shell width, (**B**) hinge length, and (**C**) nacreous height of the right valve. Points represent individual specimens, and *p*-values indicate significant differences between species.

One-way ANOVA performed on shell width, hinge length, and nacreous height of the right valve, revealed significant differences between specimens morphologically identified as *I. australicus* and *I. bicolor*. Shell width differed significantly between the two species (F_1,36_ = 4.25, *p* = 0.047, η^2^ = 0.11), as did hinge length (F_1,36_ = 5.63, *p* = 0.023, η^2^ = 0.14). The largest difference was observed for the nacreous height of the right valve (F_1_,_36_ = 28.61, *p* < 0.001, η^2^ = 0.44), indicating that this character contributed most strongly to the morphological differentiation between the two species (Figure 4).

**Figure 5:**
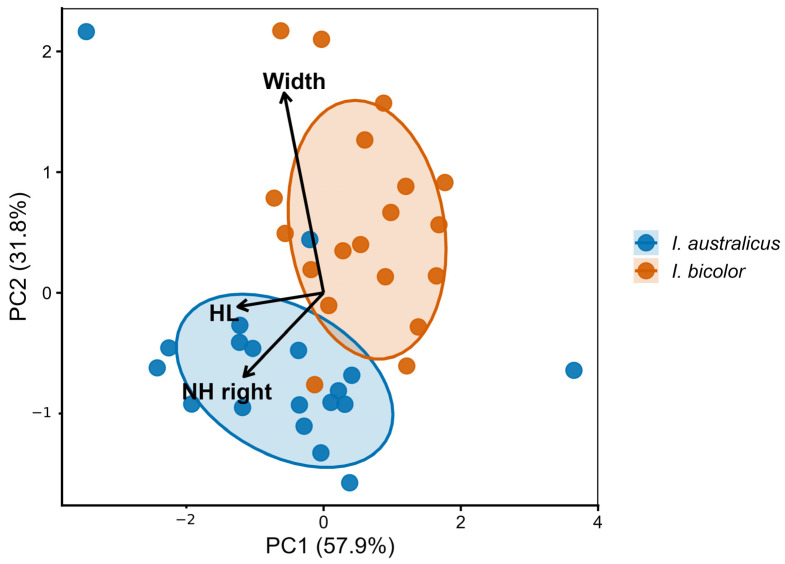
Principal Component Analysis (PCA) of shell morphometric measurements of *I. bicolor* and specimens morphologically identified as *I. australicus*. PC1 and PC2 explained 57.9% and 31.8% of the total variation, respectively. Arrows indicate the morphometric variables.

To further investigate morphological variation between species, Principal Component Analysis (PCA) was performed using shell width, hinge length, and nacreous height of the right valve (Figure 5). The first two principal components explained 89.6% of the total morphological variation (PC1 = 57.9%; PC2 = 31.8%). PC1 was primarily associated with hinge length and nacreous height, whereas PC2 was mainly influenced by shell width. The PCA showed only partial separation between specimens morphologically identified as *I. australicus* and *I. bicolor*, with considerable overlap between the two species in the ordination space (Figure 5).

#### 3.1.3. Shell Shape and Growth Patterns

**Figure 6:**
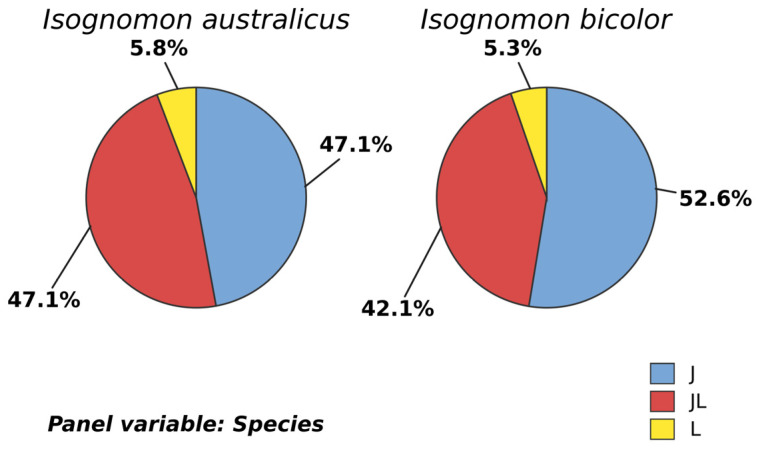
Distribution of shell growth orientation in specimens morphologically identified as *I. australicus* and *I. bicolor*. Pie charts show the percentage of specimens exhibiting posterior (J), straight (JL), and anterior (L) growth orientations.

**Figure 7:**
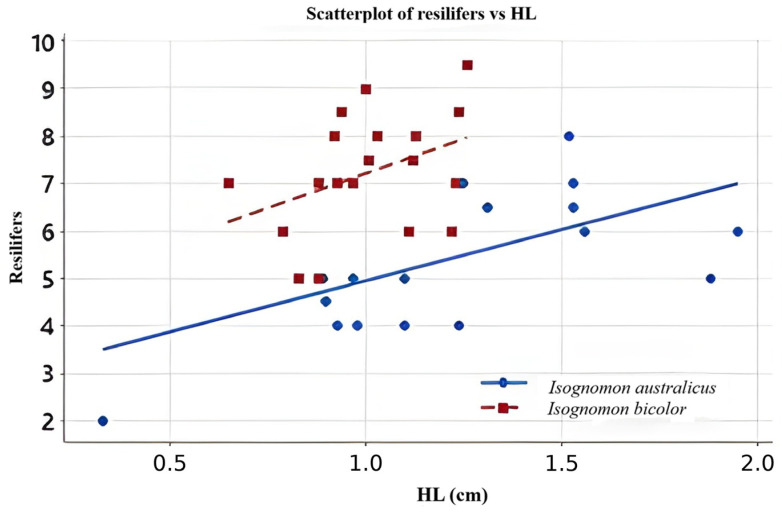
Relationship between the number of hinge resilifers and hinge length (HL) in specimens morphologically identified as *I. australicus* and *I. bicolor*. Trend lines represent the linear relationship for each species.

The vast majority of valves in both species showed posterior growth orientation (J) or no directional change (JL), in approximately equal proportions, while only a small percentage (5–6%) exhibited anterior orientation (L), corresponding to one individual per species (Figure 6). The number of hinge resilifers increased with hinge length (HL) in both *Isognomon* species (Figure 7). Specimens morphologically identified as *I. australicus* exhibited 4.3 resilifers per cm of hinge length, compared with 6.5 resilifers per cm in *I. bicolor*, corresponding to mean spacings of approximately 2.33 and 1.54 mm between successive resilifers, respectively.

#### 3.1.4. Relationships

Significant and strong relationships (R^2^ > 80%) among the measured biological parameters of the studied *Isognomon* specimens are presented in Table 2. These measurements refer to a limited number of adult individuals collected during summer 2023, which were in good body condition (Figure 2). In particular, the relationship between the nacreous layer heights of the two valves in specimens morphologically identified as *I. australicus* was significant (*p* < 0.05) and strong (R^2^ = 95.70%) (Table 2).

### 3.2. Genetics

#### 3.2.1. *16S rRNA* Analysis

Partial *16S rRNA* sequences were successfully obtained from 17 of the 20 *I. bicolor* specimens and from two of the 16 specimens morphologically identified as *I. australicus*. The 17 *I. bicolor* sequences comprised 11 distinct haplotypes (1.1, 1.5, 1.9, 1.11, 2.1, 2.2, 2.5, 3.4, 3.6, 3.7 and 3.9; Supplementary Table S1). Sequence similarity searches using BLAST showed that all *I. bicolor* haplotypes shared >98% identity with *I. bicolor* reference sequences available in GenBank. The single *I. australicus* haplotype (2.3/2.9; Supplementary Table S1) showed the highest sequence similarity to *I. legumen* and *I. aff. legumen* reference sequences available in GenBank.

**Figure 8:**
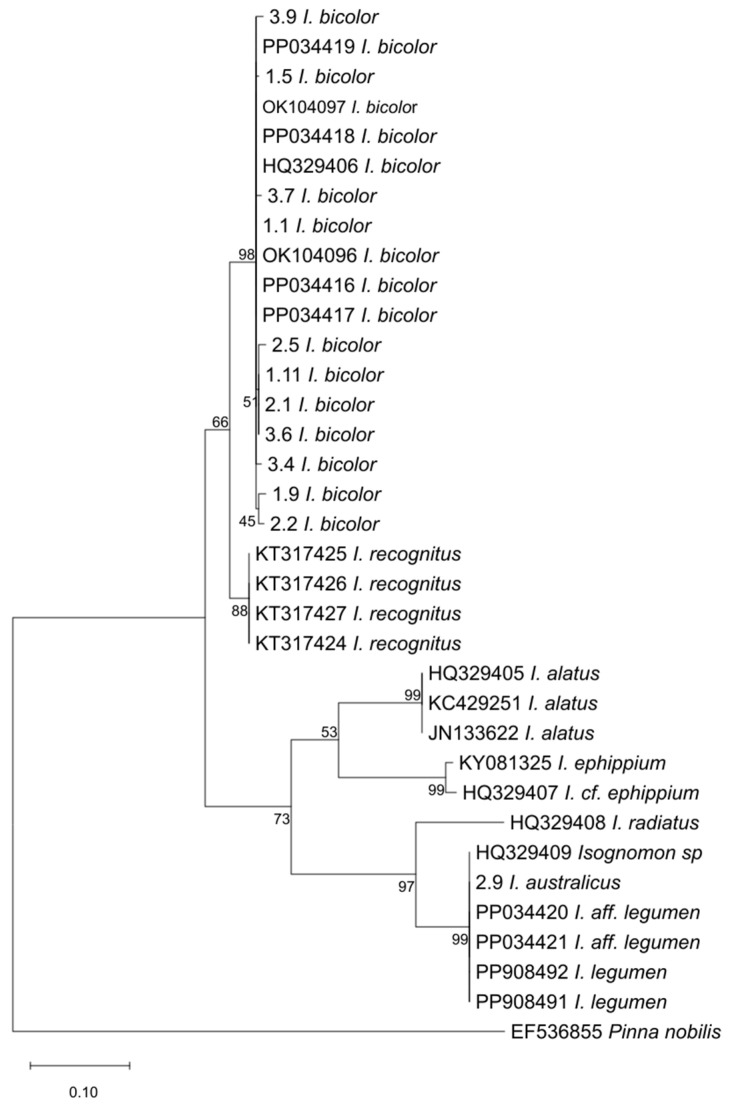
Maximum Likelihood phylogenetic tree based on the *16S rRNA* dataset of *Isognomon* spp. Numbers at the nodes indicate bootstrap support percentages based on 1000 replicates; all bootstrap values are shown. Branch lengths represent substitutions per site. *Pinna nobilis* was used as the outgroup.

Maximum Likelihood phylogenetic analysis recovered all newly identified *I. bicolor* haplotypes within a well-supported clade together with reference *I. bicolor* sequences (Figure 8). In contrast, the haplotype morphologically identified as *I. australicus* clustered with *I. legumen* and *I. aff. legumen* reference sequences and was not recovered as a distinct lineage.

The NJ analysis placed all newly identified *I. bicolor* haplotypes together with published *I. bicolor* reference sequences, with strong bootstrap support (100%; Supplementary Figure S1). The haplotype from specimens morphologically identified as *I. australicus* clustered with *I. legumen* sequences (98% bootstrap support) within the broader *I. legumen*–*I. aff. legumen* group (100% bootstrap support).

#### 3.2.2. *COI* Analysis

Partial *COI* sequences obtained from specimens morphologically identified as *I. australicus*, comprised five distinct haplotypes (2.3, 2.9, 4.2, 4.6 and 4.7; Supplementary Table S2). Sequence similarity searches using BLAST showed that all haplotypes shared >99% identity with *I. legumen* and *I. aff. legumen* reference sequences available in GenBank. Similarly, searches against the Barcode of Life Data System (BOLD) showed >99% similarity with corresponding reference records.

**Figure 9:**
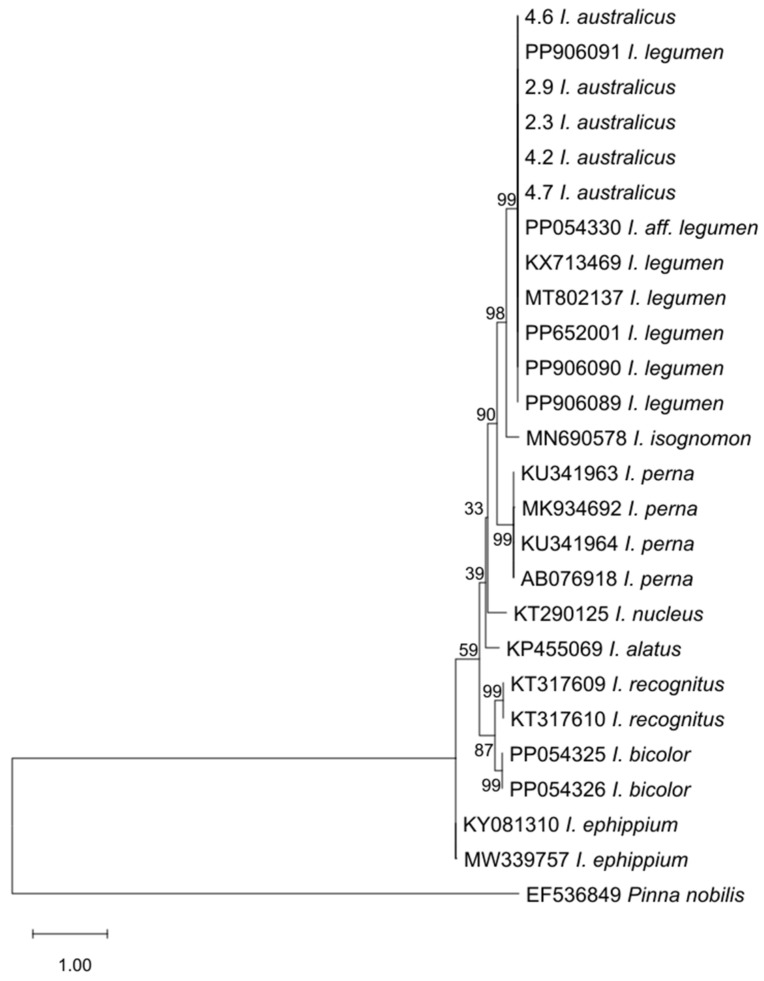
Maximum Likelihood phylogenetic tree based on the *COI* dataset of *Isognomon* spp. Numbers at the nodes indicate bootstrap support percentages based on 1000 replicates; all bootstrap values are shown. Branch lengths represent substitutions per site. *Pinna nobilis* was used as the outgroup. The outgroup branch was truncated for visualization, as indicated by the break mark.

Maximum Likelihood phylogenetic analysis recovered the five newly identified haplotypes from specimens morphologically identified as *I. australicus* within the *I. legumen–I. aff. legumen* clade, together with published reference sequences (Figure 9) and did not form a distinct lineage.

The NJ analysis placed all sequences from specimens morphologically identified as *I. australicus* together with *I. legumen* and *I. aff. legumen* reference sequences in a strongly supported cluster (99%; Supplementary Figure S2). However, the internal relationships within this cluster received low bootstrap support (47%).

#### 3.2.3. *28S rRNA* Analysis

**Figure 10:**
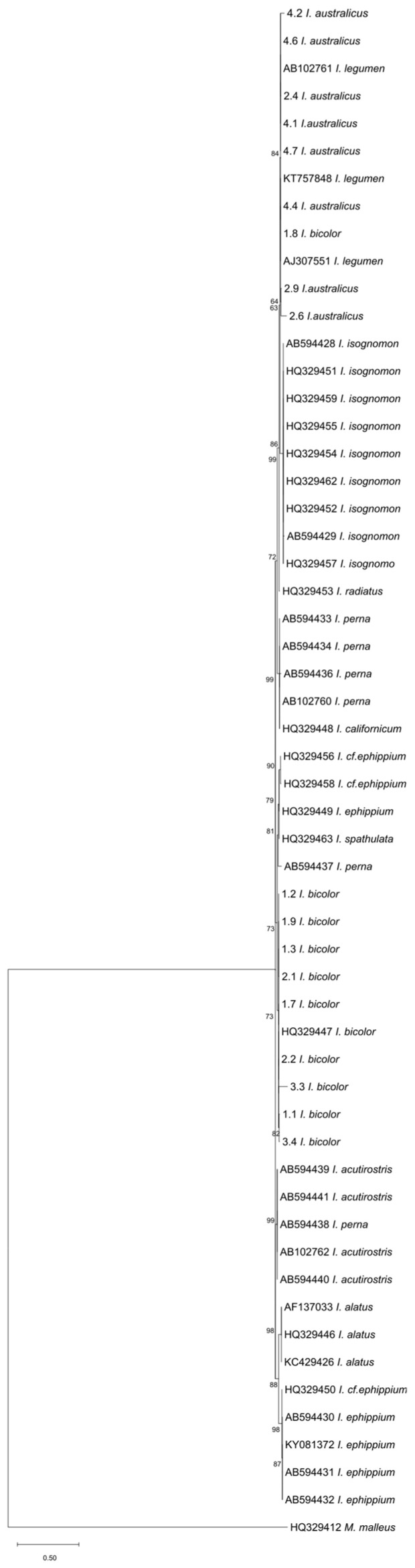
Maximum Likelihood phylogenetic tree based on the *28S rRNA* dataset of *Isognomon* spp. Numbers at the nodes indicate bootstrap support percentages based on 1000 replicates; all bootstrap values are shown. Branch lengths represent substitutions per site. *Malleus malleus* was used as the outgroup. The outgroup branch was truncated for visualization, as indicated by the break mark.

Partial *28S rRNA* sequences were obtained from specimens morphologically identified as *I. australicus* and *I. bicolor*. The *I. bicolor* sequences comprised ten distinct haplotypes, whereas the specimens morphologically identified as *I. australicus* comprised eight distinct haplotypes (Supplementary Table S3). Sequence similarity searches using BLAST identified the *I. bicolor* sequences as *I. bicolor*, whereas the sequences obtained from specimens morphologically identified as *I. australicus* showed the highest sequence similarity to *I. legumen* reference sequences available in GenBank. Maximum Likelihood phylogenetic analysis recovered the *I. bicolor* sequences within a distinct clade, together with published *I. bicolor* reference sequences (Figure 10). In contrast, sequences obtained from specimens morphologically identified as *I. australicus* clustered together with *I. legumen* reference sequences and did not form a separate lineage from *I. legumen*. Notably, haplotype 1.8, derived from a specimen morphologically identified as *I. bicolor*, was also placed within this cluster.

The NJ analysis placed the sequences from specimens morphologically identified as *I. australicus* together with *I. legumen* reference sequences, although internal support within this group was generally low (24–67%; Supplementary Figure S3). Haplotype 1.8, despite originating from a specimen morphologically identified as *I. bicolor*, was also placed within this group, whereas the remaining *I. bicolor* haplotypes clustered separately with the *I. bicolor* reference sequence.

#### 3.2.4. Comparison of Phylogenetic Methods

The NJ analyses were broadly congruent with the ML analyses across the three molecular markers (Supplementary Figures S1–S3). For the *16S rRNA* and *COI* datasets, both methods placed the sequences obtained from specimens morphologically identified as *I. australicus* with *I. legumen* and *I. aff. legumen* reference sequences, while the *16S rRNA* sequences of *I. bicolor* clustered with the corresponding reference sequences. Similar overall relationships were recovered for the *28S rRNA* dataset. However, haplotype 1.8, derived from a specimen morphologically identified as *I. bicolor*, was separated from the remaining *I. bicolor* sequences and clustered with the morphologically identified *I. australicus* specimens and *I. legumen* references in both the ML and NJ trees (Figure 10; Supplementary Figure S3). This incongruence may reflect morphological misidentification of the specimen or the limited discriminatory power of the slowly evolving *28S rRNA* marker among closely related *Isognomon* taxa. Other differences between the methods were primarily restricted to weakly supported internal relationships and did not alter the principal taxonomic placement of the examined specimens.

#### 3.2.5. Genetic Distance Analysis

Pairwise genetic distance analyses were consistent with the phylogenetic relationships recovered by the Maximum Likelihood analyses (Table 3). The *16S rRNA* and *28S rRNA* sequences obtained from *I. bicolor* exhibited low genetic divergence from published *I. bicolor* reference sequences. Additionally, specimens morphologically identified as *I. australicus* showed the lowest genetic distances to *I. legumen* and *I. aff. legumen* reference sequences, supporting their placement within the *I. legumen–I. aff. legumen* complex. Complete pairwise genetic distance matrices for each molecular marker are provided in Supplementary Tables S1–S3.

#### 3.2.6. Genetic Diversity

Genetic diversity based on the *28S rRNA* marker is summarized in Table 4. Among specimens morphologically identified as *I. australicus*, Agia Pelagia comprised five haplotypes among eight specimens, exhibiting high haplotype diversity (Hd = 0.857), a nucleotide diversity of 0.00781, and 19 segregating sites. In Kissamos, each of the four specimens represented a unique haplotype (H = 4, Hd = 1.000), with a nucleotide diversity of 0.02229 and 28 segregating sites. The two specimens from Ierapetra shared a single haplotype and showed no genetic variation (Hd = 0.000, π = 0.00000, S = 0).

For *I. bicolor*, Agia Pelagia comprised six haplotypes among ten specimens (Hd = 0.778), with a nucleotide diversity of 0.01303 and 43 segregating sites. Kissamos contained three haplotypes among four specimens, resulting in high haplotype diversity (Hd = 0.833), a nucleotide diversity of 0.00467, and 11 segregating sites. Ierapetra comprised three haplotypes among six specimens and exhibited the highest nucleotide diversity (π = 0.02205) and greatest number of segregating sites (S = 45), despite showing moderate haplotype diversity (Hd = 0.600).

#### 3.2.7. *COI* Haplotype Network

**Figure 11:**
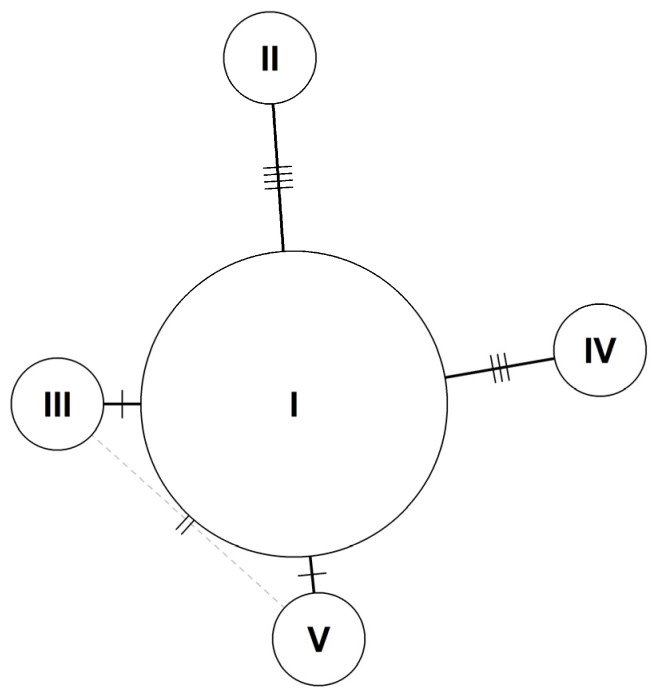
*COI* haplotype network of specimens morphologically identified as *I. australicus*. Circle size is proportional to the number of individuals sharing each haplotype, and hatch marks represent mutational steps between haplotypes. Solid lines represent the primary parsimonious connections between haplotypes, whereas the dotted line indicates an alternative equally parsimonious connection.

The *COI* haplotype network identified **five haplotypes** among the analyzed specimens (Figure 11). Haplotype I was the most common and occupied a central position in the network, whereas Haplotypes II–V were less frequent. Haplotype III and Haplotype V differed from Haplotype I by a single mutational step, while Haplotypes II and IV were separated from the dominant haplotype by multiple mutational steps. Overall, the network exhibited a star-like topology, with the most frequent haplotype connected to several less common derived haplotypes.

## 4. Discussion

### 4.1. Ecological Information and Biotope

Crete, the southernmost island of Greece, experiences warm sea surface temperatures during the summer months. At the three sampling locations examined in the present study (Kissamos, Agia Pelagia, and Ierapetra), the mean August sea surface temperature for the period 2022–2025 was approximately 26 °C. During the sampling period (August 2023), sea surface temperatures ranged from 25–28 °C in Kissamos, 26–28 °C in Agia Pelagia, and 24–27 °C in Ierapetra (Web Resource 7). These temperature conditions fall within the range reported for established populations of tropical *Isognomon* species and may facilitate their persistence and establishment in the Mediterranean Sea [7,8]. Similarly, the successful establishment of the alien *I. bicolor* in Brazilian waters [21,38] has been associated with temperatures ranging from 25.6–28.7 °C annually and 27.7–29.8 °C during the warmest months (Web Resource 8), closely resembling those recorded at the Cretan sampling sites. Elevated sea surface temperatures have been suggested to promote fertilization success and larval development in sessile bivalves, thereby facilitating the establishment of non-indigenous molluscan species in the Mediterranean Sea [9,24,53,54,55].

In addition to favorable temperatures, relatively high salinity levels have been reported for the southern Aegean and Cretan Seas, with values exceeding 39‰ in certain water masses and periods [56]. Although salinity varies spatially, seasonally, and with depth, these values are consistent with the hypersaline conditions under which *I. bicolor* has been reported in Brazil, where the species tolerates salinity levels ranging from 38 to 50‰ [21]. This broad salinity tolerance suggests a high degree of environmental adaptability that may contribute to the persistence and spread of the species in non-native regions. Experimental evidence also indicates that *I. bicolor* is more tolerant to salinity stress than several other bivalve species [57]. In Brazil, the species has been reported mainly from intertidal rocky shores and hypersaline estuaries, including the Casqueira and Tubarão rivers [21]. Our observations of established populations on rocky substrates in Crete are consistent with the ecological flexibility previously reported for this species. Furthermore, the coexistence of the two studied *Isognomon* taxa within the same localities suggests that both can persist under similar environmental conditions. 

Based on the limited availability of published ecological and biological information for *I. australicus*, interpretation of its environmental tolerance and establishment dynamics in the Mediterranean Sea remains constrained. Among species of the genus *Isognomon,* research attention has focused primarily on *I. ephippium*, followed by *I. bicolor*, *Isognomon isognomon*, and *I. alatus*, whereas no recent publications were identified for several other species, including *I. australicus* [18]. This lack of species-specific information restricts direct comparisons with previously studied populations and highlights the importance of documenting new occurrence data such as those provided in the present study, to improve understanding of its distribution and ecological requirements within newly colonized regions.

### 4.2. Biometry

The biometric characteristics of *I. bicolor* individuals from Crete, particularly hinge length (HL), number and density of resilifers, and nacre height (NH), are consistent with values previously reported in the literature [22,38,39]. In contrast, biometric information for *I. australicus* remains limited, as the species has only recently been reported in the Eastern Mediterranean, primarily based on juvenile individuals [24]. The specimens examined in the present study were adults, and their measurements provide, for the first time, representative biometric data for the species in this region during the sampling period, thereby expanding the currently limited morphological information available for the Eastern Mediterranean.

The one-way ANOVA demonstrated significant differences between specimens morphologically identified as *I. australicus* and *I. bicolor* for shell width, hinge length, and the nacreous height of the right valve. Among these variables, the nacreous height exhibited the strongest differentiation between species (η^2^ = 0.44), suggesting that the relative development of the nacreous layer represents one of the most consistent shell characters distinguishing adult specimens. In contrast, shell width and hinge length exhibited smaller, although still significant, differences between species.

Principal Component Analysis (PCA) further supported these observations by revealing only partial separation between the specimens morphologically identified as *I. australicus* and *I. bicolor*. Although the first two principal components explained almost 90% of the total morphological variation, considerable overlap between species remained evident, reflecting the pronounced shell plasticity that has long been recognized within the genus *Isognomon* [18,32]. These findings indicate that combinations of the examined shell measurements improve morphological differentiation, but do not provide complete separation between the two species.

### 4.3. Shape and Growth

Shell morphology in both species appears to be strongly influenced by habitat conditions, including the presence of crevices or cavities, hydrodynamic regime, and population density [21,22]. Individuals inhabiting crevices or narrow cavities may develop elongated shells due to spatial constraints, while shell size and shape can vary among sites depending on local environmental conditions and substrate structure [21,28,38,58]. Similar habitat-related variability has been reported from intertidal rocky substrates and oyster banks, where individuals frequently occupy rock crevices or dense aggregations that influence valve elongation and overall shell morphology [21,39].

This environmental influence is reflected in the pronounced shell plasticity observed in the present study. Adult specimens displayed considerable morphological variability, while shell orientation followed a similar pattern in both species, with approximately half of the examined individuals presenting rotated shells and the remaining half presenting straight shells.

Apart from shell shape, individual size is also influenced by age and seasonal timing [59]. Differences between the two species were also observed in hinge morphology. Although hinge length differed significantly between species, *I. bicolor* generally presented a greater number of resilifers relative to hinge length than specimens morphologically identified as *I. australicus*. This structural difference may reflect differences in ecological adaptation between the two species, although the functional significance of resilifer number remains poorly understood and warrants further investigation.

### 4.4. Distribution and Genetic Evidence of Specimens Morphologically Identified as I. australicus

The Indo-Pacific species *I. australicus* has been increasingly reported in the Eastern Mediterranean, including records from Astypalaia (Greece) [23] (*I. australica*), Crete and Cyprus [24] (*I. aff. australica*), Attica (Greece) [25] (*I. australica*), Preveza (Greece) [6] (*I. australicus*), and Lefkada (Greece) [60] (*I. aff. australica*). Additionally, *I. australicus* was previously misclassified as *I. legumen* in Mediterranean records, while *I. legumen* has also been reported as *I. australicus* (Reeve, 1858) from the Ionian Islands of Greece and as *I. aff. australica* from southern Crete and Cyprus [28].

Phylogenetic analyses based on the mitochondrial (*16S rRNA* and *COI*) and nuclear (*28S rRNA*) markers consistently recovered the specimens morphologically identified as *I. australicus* within a mixed clade comprising *I. legumen* and *I. aff. legumen*. This pattern agrees with previous studies reporting an unresolved relationship among these taxa and supports the view that their taxonomy remains uncertain [7,28]. Similarly, the genetic distance analyses showed that the examined specimens were genetically closest to *I. legumen* and *I. aff. legumen*, irrespective of the molecular marker analyzed, further supporting their placement within the *I. legumen*–*I. aff. legumen* complex. Although the examined specimens were consistently placed within the *I. legumen–I. aff. legumen* complex, several internal nodes showed low bootstrap support, particularly in the *28S rRNA* tree. This indicates limited phylogenetic resolution among closely related lineages, likely due to low sequence divergence and the limited availability of well-characterized reference sequences. Although *28S rRNA* provided independent nuclear evidence, its relatively slow evolutionary rate may limit its ability to resolve closely related or recently diverged *Isognomon* lineages. Therefore, the 28S results should be interpreted as complementary evidence rather than definitive species-level discrimination.

Nevertheless, the concordant results across the three markers support their affinity with the *I. legumen–I. aff. legumen* complex. The concordance between the mitochondrial and nuclear datasets suggests that the observed pattern is not solely attributable to the use of a single molecular marker. Instead, it most likely reflects unresolved taxonomy, limitations of the currently available reference datasets, or the existence of a closely related species complex within the genus *Isognomon* [13,28]. Additional integrative taxonomic studies combining broader geographic sampling, multiple independent nuclear markers, and comprehensive morphological analyses will be required to clarify the taxonomic boundaries among these taxa.

The inclusion of the nuclear *28S rRNA* marker provided an independent assessment of species identity and yielded phylogenetic relationships consistent with those obtained from the mitochondrial datasets. Although the additional marker did not improve the resolution of the *I. australicus*–*I. legumen* complex, it independently corroborated the observed clustering pattern, suggesting that the remaining uncertainty reflects the current taxonomic status of this species complex rather than insufficient information from a single mitochondrial locus.

### 4.5. Distribution and Molecular Confirmation of I. bicolor

*I. bicolor*, originating from the Caribbean region [18], has become a widespread invasive species along the Brazilian coast. In the Mediterranean, it was historically misidentified as *I. legumen* (Table 1 in [13]) until its presence was confirmed using molecular evidence [26]. Subsequent studies employing additional genetic markers further supported its identification and documented its continued spread within the Mediterranean basin [7,13].

In the present study, all newly generated *16S rRNA* sequences and all *28S rRNA* sequences, except haplotype 1.8, clustered consistently with published *I. bicolor* reference sequences, while genetic distance analyses revealed very low divergence from authenticated reference material. Haplotype 1.8, derived from a specimen morphologically identified as *I. bicolor*, instead clustered with the morphologically identified *I. australicus* specimens and *I. legumen* reference sequences, potentially reflecting morphological misidentification or the limited discriminatory power of the *28S rRNA* marker. However, because of the limited sample size, broader phylogeographic conclusions cannot be drawn. Collectively, the morphological observations and the concordant mitochondrial and nuclear evidence provide strong support for the identification of *I. bicolor* in Crete.

### 4.6. Genetic Diversity Patterns

The genetic diversity analyses were consistent with the phylogenetic and haplotype network results, revealing low to moderate levels of genetic variation among the examined specimens. *I. australicus* exhibited high haplotype diversity in Agia Pelagia and Kissamos, with the latter showing a unique haplotype for each analyzed individual despite the limited sample size. In contrast, *I. bicolor* displayed moderate haplotype diversity across the three locations, with the highest nucleotide diversity and number of segregating sites recorded in Ierapetra, indicating greater sequence divergence among the detected haplotypes. Overall, the combination of low pairwise genetic distances, moderate nucleotide diversity, and the distribution of haplotypes supports the presence of closely related lineages within both taxa, while indicating geographic variation in genetic diversity among the sampled populations.

### 4.7. Introduction Pathways and Dispersal Mechanisms

The introduction and dispersal of tropical marine species into the Mediterranean Sea may involve multiple pathways, including both natural range expansions and human-mediated transport [7,61]. Although the Suez Canal is considered a major introduction corridor for Indo-Pacific species, the occurrence of *I. bicolor*, native to the tropical western Atlantic/Caribbean region, has been associated with west-to-east Atlantic transport and anthropogenic vectors [26,28]. Previous studies have suggested that transport through the Strait of Gibraltar may have been facilitated by changing oceanographic conditions and hydrographic circulation patterns [7,62], which could contribute to larval transport toward suitable habitats in the eastern Mediterranean [23,62]. Furthermore, the warm sea surface temperatures recorded at the Cretan sampling sites during the study period are comparable to those reported from regions where *I. bicolor* has successfully established [21,38], potentially supporting its persistence following introduction.

The first record of *I. bicolor* in Crete reported herein is consistent with the ongoing eastward expansion of the species within the Mediterranean Sea. However, the precise pathway of introduction cannot be determined from the available data. Commercial shipping has frequently been proposed as one of the most likely vectors for the introduction and spread of *I. bicolor*, particularly through ballast water discharge and hull fouling [28,38,63]. Other pathways, including the relocation of offshore platforms [20,38] and rafting on floating debris or macroalgae [7,21,64,65], have also been suggested. Following introduction, secondary spread may occur through planktonic larval dispersal facilitated by regional hydrodynamic circulation and habitat connectivity [28,66,67]. The occurrence of established populations on rocky substrates near coastal areas of Crete is compatible with these previously proposed introduction and dispersal mechanisms.

The successful establishment of both *I. bicolor* and specimens morphologically identified as *I. australicus* in the study area is likely supported by increasing sea surface temperatures associated with global warming, which create favorable environmental conditions for the persistence of thermophilic non-indigenous species in the Eastern Mediterranean. However, their introduction pathways into the region appear to differ. The occurrence of specimens morphologically identified as *I. australicus* in Greek waters may be associated either with Lessepsian migration through the Suez Canal or with shipping-related transport, whereas the introduction of *I. bicolor* is more likely linked primarily to vessel-mediated dispersal. The co-occurrence of the two species in the study area further highlights the role of the Eastern Mediterranean, and particularly Crete, as an active zone of establishment for non-indigenous *Isognomon* species arriving through multiple dispersal mechanisms.

## 5. Conclusions

The present study confirms the establishment of the non-indigenous oyster *I. bicolor* along the coasts of Crete (Greece) and documents the presence of specimens belonging to the unresolved *I. australicus–I. legumen–I. aff. legumen* complex. Both taxa were recorded from similar rocky coastal habitats, indicating their successful establishment and coexistence under comparable environmental conditions in the eastern Mediterranean Sea.

Morphometric analyses of adult specimens revealed pronounced shell plasticity and identified shell width, hinge length, and nacreous height as the most informative characters for distinguishing the examined taxa. However, the substantial overlap in shell morphology demonstrates that external shell characters alone remain insufficient for reliable species identification, highlighting the limitations of morphology-based taxonomy within the genus *Isognomon*.

Molecular analyses provided strong support for the identification of *I. bicolor* and further documented its eastward expansion within the Mediterranean basin. In contrast, both the mitochondrial (*COI* and *16S rRNA*) and nuclear (*28S rRNA*) datasets consistently placed the *I. australicus*-like specimens within the *I. legumen–I. aff. legumen* complex. The concordance between mitochondrial and nuclear markers indicates that the observed taxonomic uncertainty is unlikely to result solely from the use of a single molecular marker. Instead, it likely reflects unresolved species boundaries, limitations of the currently available reference datasets, or the presence of a closely related species complex within the genus.

By integrating adult morphometric analyses with mitochondrial and nuclear genetic data, this study expands the available knowledge of *Isognomon* populations in the eastern Mediterranean and provides additional evidence supporting the need for a comprehensive taxonomic revision of the *I. australicus–I. legumen–I. aff. legumen* complex. Future studies incorporating broader geographic sampling, well-documented reference material from the native distribution ranges, and additional nuclear or genomic markers will be essential for resolving species boundaries and clarifying evolutionary relationships within this taxonomically challenging group. Continued monitoring of Mediterranean *Isognomon* populations will also improve our understanding of their distribution, establishment dynamics, and potential ecological implications under ongoing environmental change.

**Web Resources:**

1. WoRMS Editorial Board. World Register of Marine Species. Available online: https://www.marinespecies.org (accessed on 19 July 2026).
2. IDSCARO. Italian DNA Barcode Reference Library for Marine Organisms. Available online: https://www.idscaro.net (accessed on 19 July 2026).
3. CMCC Foundation. Over 30 °C: Marine Heatwaves Currently Hitting the Mediterranean Sea. available online: https://www.cmcc.it/article/over-30c-marine-heatwaves-currently-hitting-the-mediterranean (accessed on 19 July 2026).
4. The Seashells of New South Wales. Isognomonidae. Available online: https://seashellsofnsw.org.au/Isognomonidae/Pages/Isognomonidae_Intro.htm (accessed on 19 July 2026).
5. National Center for Biotechnology Information (NCBI). Basic Local Alignment Search Tool (BLAST). Available online: https://blast.ncbi.nlm.nih.gov/Blast.cgi (accessed on 19 July 2026).
6. Barcode of Life Data Systems (BOLD). Available online: https://www.boldsystems.org (Accessed on 19 July 2026).
7. SeaTemperature.net. Water Temperature Worldwide: Seas, Lakes and Rivers. Available online: https://seatemperature.net/ (accessed on 19 July 2026).
8. SeaTemperature.org. Brazil Sea Temperature. Available online: https://www.seatemperature.org/south-america/brazil (accessed on 19 July 2026).

## Figures and Tables

**Table 1:** Mean ± StDev values of biometrical parameters for *I. bicolor* and morphologically identified as *I. australicus.* N: number of specimens.

Variable	Morphologically Identified as *I. australicus*		*I. bicolor*	
	N	Mean ± StDev	N	Mean ± StDev
Length (cm)	19	2.11 ± 0.46	21	1.67 ± 0.74
Height (cm)	19	3.53 ± 1.12	21	2.25 ± 0.84
Width (cm)	19	0.52 ± 0.15	21	0.57 ± 0.21
Total wet weight (g)	19	1.86 ± 0.83	20	1.16 ± 0.73
Body wet weight (g)	19	0.21 ± 0.12	19	0.13 ± 0.07
Valves wet weight (g)	19	1.56 ± 0.67	19	1.21 ± 1.00
Hinge length (HL, cm)	19	1.26 ± 0.43	19	1.01 ± 0.17
Resilifers	18	5.42 ± 1.58	21	6.55 ± 2.48
Nacreous height of left valve (NH left, cm)	19	2.57 ± 0.59	19	1.21 ± 0.36

**Table 2:** The most significant and strong relationships (R^2^ > 80%) of the two studied species.

Species	N	Equation	*p* (ANOVA)	R^2^%
*I. bicolor*	13	log_10_(NH left) = 0.07504 + 1.338 log_10_(HL)	<0.001	95.99
Specimens morphologically identified as *I. australicus*	14	log_10_(NHleft)= 0.1385 + 0.9800 log_10_(NHright)	<0.001	95.70
	12	log_10_(resilifers) = 0.6609 + 0.6957 log_10_(HL)	<0.001	85.01

**Table 3:** Summary of genetic distances between the examined *Isognomon* specimens and reference species based on the *16S rRNA*, *COI*, and *28S rRNA* datasets. Complete marker-specific datasets were included in the analyses. Values represent the mean pairwise genetic distances between all specimens analyzed in this study (in the indicated morphological group) and the corresponding reference sequences.

Marker	Specimens Analyzed in This Study	Reference Species	Genetic Distance
*16S rRNA*	*I. bicolor*	*I. bicolor*	0.00499
*16S rRNA*	*I. australicus*	*I. legumen*	0.00000
*16S rRNA*	*I. australicus*	*I. aff. legumen*	0.00456
*COI*	*I. australicus*	*I. legumen*	0.00100
*COI*	*I. australicus*	*I. aff. legumen*	0.00100
*28S rRNA*	*I. bicolor*	*I. bicolor*	0.01512
*28S rRNA*	*I. australicus*	*I. legumen*	0.03681

**Table 4:** Genetic diversity indices of specimens morphologically identified as *I. australicus* and *Isognomon bicolor* based on *28S rRNA* sequences from the three sampling locations in Crete. Diversity indices were not calculated for specimens morphologically identified as *I. australicus* from Ierapetra because only one sequence was available.

Species	Location	N	H	Hd	π	S
morphologically identified as *I. australicus*	Agia Pelagia	8	5	0.857	0.00781	19
morphologically identified as *I. australicus*	Kissamos	4	4	1.000	0.02229	28
morphologically identified as *I. australicus*	Ierapetra	2	1	**-**	-	-
*I. bicolor*	Agia Pelagia	10	6	0.778	0.01303	43
*I. bicolor*	Kissamos	4	3	0.833	0.00467	11
*I. bicolor*	Ierapetra	6	3	0.600	0.02205	45

Abbreviations: N = number of sequences; H = number of haplotypes; Hd = haplotype diversity; π = nucleotide diversity; S = number of segregating sites.

## Data Availability

The DNA sequences generated in this study have been submitted to GenBank. The currently available accession numbers are PQ638911–PQ638921, PV166450–PV166454 and PX915791. Accession numbers for the *28S rRNA* sequences are pending and will be added before final publication. Additional data supporting the findings of this study are available within the article and its Supplementary Materials.

## Supplementary Materials

The following supporting information can be downloaded at: https://www.mdpi.com/article/10.3390/ani16142277/s1, Table S1: ΜtDNA *16S rRNA* sequences used for the phylogenetic analysis in the present study. Table S2: ΜtDNA *COI* sequences used for the phylogenetic analysis in the present study. Table S3: *28S rRNA* sequences used for the phylogenetic analysis in the present study. Figure S1: Neighbor-Joining phylogenetic tree based on the *16S rRNA* dataset. Figure S2: Neighbor-Joining phylogenetic tree based on the *COI* dataset. Figure S3: Neighbor-Joining phylogenetic tree based on the *28S rRNA* dataset.
