# Supplementary material for: New Records of Isognomon Species from Crete, Greece; Evidence from Adult Specimens and Additional DNA Barcodes
Source: Animals (Basel). 2026 Jul 22;16(14):2277. doi: 10.3390/ani16142277 (PMC13405694; doi:10.3390/ani16142277)
Supplement: Supplementary file 1 [file animals-16-02277-s001.zip › Supplementary Table S1.pdf]

Table S1. MtDNA 16S rRNA sequences used for the phylogenetic analysis in the present study.

**Specimens/ Species**	**GenBank Accession numbers**	**References**
1.1, 1.5, 1.9, 1.11, 2.1, 2.2, 2.5, 3.4, 3.6, 3.7, 3.9 – *I. bicolor*	PQ638911-PQ638921	This study
2.3/2.9– *I. australicus*	PX915791	This study
I. alatus	KC429251.1	Sharma *et al*. (2013)
I. alatus	HQ329405.1	Tëmkin (2010)
I. aff. legumen	PP034421.1	Albano *et al*. (2024)
I. aff. legumen	PP034420.1	Albano *et al*. (2024)
I. legumen	PP908492.1	Angelidis *et al*. (2025)
I. legumen	PP908491.1	Angelidis *et al*. (2025)
I. bicolor	HQ329406.1	Tëmkin (2010)
I. bicolor	PP034418.1	Albano *et al*. (2024)
I. bicolor	PP034417.1	Albano *et al*. (2024)
I. bicolor	PP034416.1	Albano *et al*. (2024)
I. ephippium	KY081325.1	Liu *et al*. (2018)
I. radiatus	HQ329408.1	Tëmkin (2010)
I. recognitus	KT317427.1	Raith *et al*. (2015)
I. recognitus	KT317426.1	Raith *et al*. (2015)
Pinna nobilis	EF536855.1	Katsares *et al*. (2008)
I. bicolor	PP034419.1	Albano et al. (2024)
I. recognitus	KT317424.1	Raith et al. (2015)
I. recognitus	KT317425.1	Raith et al. (2015)
I. cf. ephippium	HQ329407.1	Tëmkin (2010)
I. alatus	JN133622.1	Unpublished, Kappner et al.
Isognomon sp.	HQ329409.1	Tëmkin (2010)
