# Supplementary material for: New Records of Isognomon Species from Crete, Greece; Evidence from Adult Specimens and Additional DNA Barcodes
Source: Animals (Basel). 2026 Jul 22;16(14):2277. doi: 10.3390/ani16142277 (PMC13405694; doi:10.3390/ani16142277)
Supplement: Supplementary file 1 [file animals-16-02277-s001.zip › Supplementary Table S2.pdf]

Table S2. MtDNA COI sequences used for the phylogenetic analysis in the present study.

**Specimens/ Species**	**GenBank Accession numbers**	**References**
2.3, 2.9, 4.2, 4.6, 4.7 - *I. australicus*	PV166450–PV166454	This study
I. alatus	KP455069.1	Pagenkopp Lohan *et al.* (2015)
I. aff. legumen	PP054330.1	Albano *et al*. (2024)
I. bicolor	PP054326.1	Albano *et al*. (2024)
I. bicolor	PP054325.1	Albano *et al*. (2024)
I. ephippium	MW339757.1	Benthotage (2022)
I. ephippium	KY081310.1	Liu *et al*. (2018)
I. legumen	MT802137.1	Patoka *et al.* (2020)
I. legumen	PP652001.1	McIlroy *et al.* (2024)
I. legumen	KX713469.1	Combosch *et al.* (2016)
I. legumen	PP906089.1	Angelidis *et al.* (2024)
I. legumen	PP906090.1	Angelidis *et al.* (2024)
I. legumen	PP906091.1	Angelidis *et al.* (2024)
I. nucleus	KT290125.1	Ardura *et al*. (2015)
I. recognitus	KT317610.1	Raith *et al.*(2015)
I. recognitus	KT317609.1	Raith *et al*. (2015)
Pinna nobilis	EF536849.1	Katsares *et al*. (2008)
I. isognomon	MN690578.1	Unpublished
I. perna	MK934692.1	Unpublished
I. perna	KU341963.1	Ardura et al. (2015)
I. perna	KU341964.1	Ardura et al. (2015)
I. perna	AB076918.1	Matsumoto (2003)
