# Supplementary material for: New Records of Isognomon Species from Crete, Greece; Evidence from Adult Specimens and Additional DNA Barcodes
Source: Animals (Basel). 2026 Jul 22;16(14):2277. doi: 10.3390/ani16142277 (PMC13405694; doi:10.3390/ani16142277)
Supplement: Supplementary file 1 [file animals-16-02277-s001.zip › Supplementary Table S3.pdf]

Specimens/Species	GenBank Accession numbers	References
1.1, 1.2, 1.3, 1.7, 1.8, 1.9, 2.1, 2.2, 3.3, 3.4 - *I. bicolor*	Accession numbers pending	This study
2.4, 2.9, 2.6, 4.1, 4.2, 4.4, 4.6, 4.7 – *I. australicus*	Accession numbers pending	This study
I. legumen	AJ307551.1	Hammer (unpublished)
I. legumen	KT757848.1	Combosch et al. (2017)
I. legumen	AB102761.1	Tëmkin (2006)
I. radiatus	HQ329453.1	Tëmkin (2010)
I. isognomon	HQ329451.1	Tëmkin (2010)
I. isognomon	HQ329452.1	Tëmkin (2010)
I. isognomon	HQ329454.1	Tëmkin (2010)
I. isognomon	HQ329455.1	Tëmkin (2010)
I. isognomon	HQ329457.1	Tëmkin (2010)
I. isognomon	HQ329459.1	Tëmkin (2010)
I. isognomon	HQ329462.1	Tëmkin (2010)
I. isognomon	AB594428.1	Matsumoto (2011)
I. isognomon	AB594429.1	Matsumoto (2011)
I. perna	AB102760.1	Tëmkin (2006)
I. perna	AB594433.1	Matsumoto (2011)
I. perna	AB594434.1	Matsumoto (2011)
I. perna	AB594436.1	Matsumoto (2011)
I. perna	AB594438.1	Matsumoto (2011)
I. californicum	HQ329448.1	Tëmkin (2010)
I. alatus	AF137033.1	Hammer (unpublished)
I. alatus	KC429426.1	Pagenkopp Lohan et al. (2015)
I. alatus	HQ329446.1	Tëmkin (2010)
I. cf. ephippium	HQ329450.1	Tëmkin (2010)
I. ephippium	KY081372.1	Liu et al. (2018)
I. ephippium	HQ329449.1	Tëmkin (2010)
I. ephippium	AB594430.1	Matsumoto (2011)
I. ephippium	AB594431.1	Matsumoto (2011)
I. ephippium	AB594432.1	Matsumoto (2011)
I. acutirostris	AB102762.1	Tëmkin (2006)
I. acutirostris	AB594439.1	Matsumoto (2011)
I. acutirostris	AB594440.1	Matsumoto (2011)
I. acutirostris	AB594441.1	Matsumoto (2011)
Malleus malleus	HQ329412.1	Tëmkin (2010)

Table S3. 28S rRNA sequences used for the phylogenetic analysis in the present study.
