# Supplementary figures and images for: New Records of Isognomon Species from Crete, Greece; Evidence from Adult Specimens and Additional DNA Barcodes
Source: Animals (Basel). 2026 Jul 22;16(14):2277. doi: 10.3390/ani16142277 (PMC13405694; doi:10.3390/ani16142277)

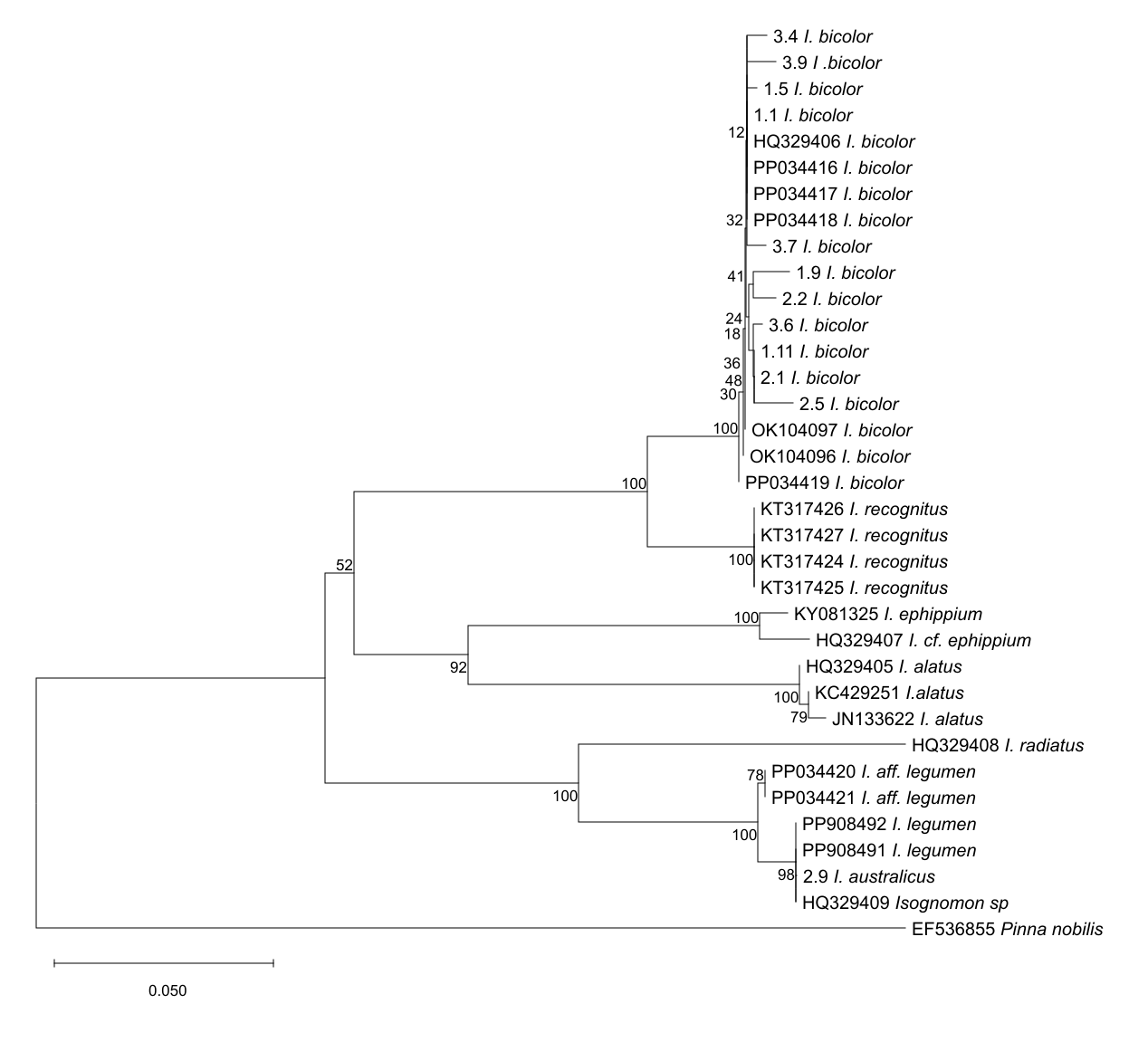

Supplement: Supplementary file 1 [file animals-16-02277-s001.zip › Supplementary Figure S1.tiff]

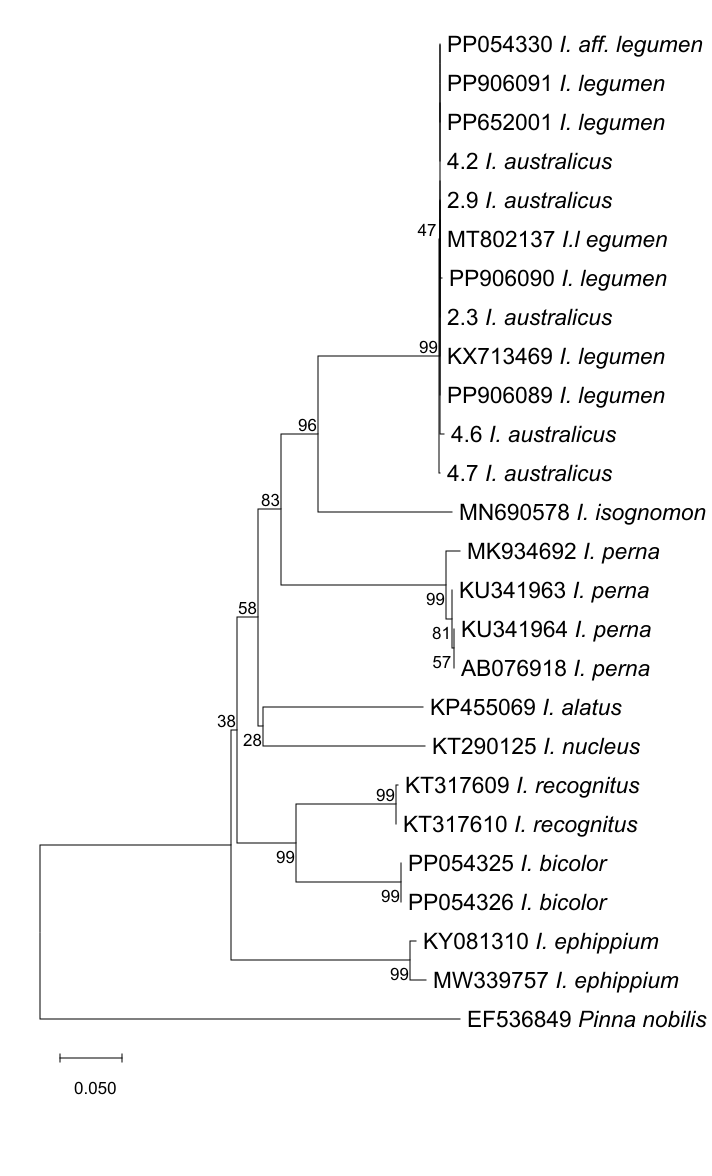

Supplement: Supplementary file 1 [file animals-16-02277-s001.zip › Supplementary Figure S2.tiff]

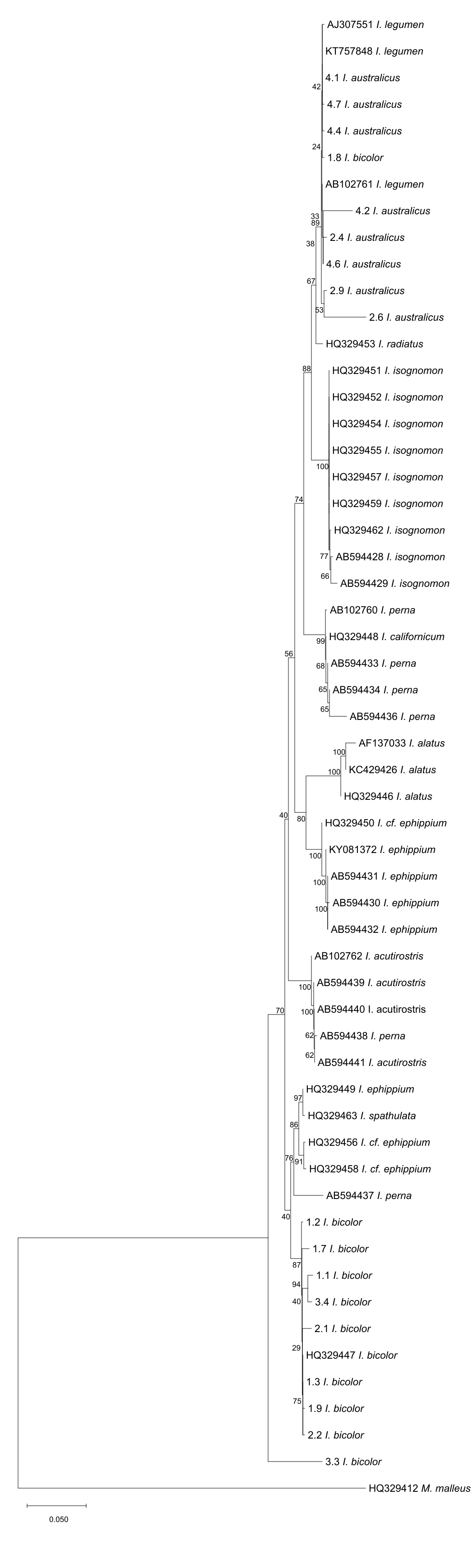

Supplement: Supplementary file 1 [file animals-16-02277-s001.zip › Supplementary Figure S3.tiff]
